# Modulation effects of different treatments on periaqueductal gray resting state functional connectivity in knee osteoarthritis knee pain patients

**DOI:** 10.1111/cns.14153

**Published:** 2023-03-08

**Authors:** Jun Zhou, Fang Zeng, Shirui Cheng, Xiaohui Dong, Nannan Jiang, Xinyue Zhang, Chenjian Tang, Wenhua He, Yang Chen, Ning Sun, Yuanfang Zhou, Xinling Li, Shengjie Hu, Ruirui Sun, Max Wintermark, Weihua Yang, Fanrong Liang, Zhengjie Li

**Affiliations:** ^1^ Chengdu University of Traditional Chinese Medicine Chengdu China; ^2^ Acupuncture & Brain Research Center Chengdu University of Traditional Chinese Medicine Chengdu China; ^3^ Hospital of Chengdu University of Traditional Chinese Medicine Chengdu China; ^4^ The Second Affiliated Hospital of Shanxi University of Traditional Chinese Medicine Taiyuan China; ^5^ Rehabilitation Medicine Center and Institute of Rehabilitation Medicine West China Hospital, Sichuan University Chengdu China; ^6^ Key Laboratory of Rehabilitation Medicine in Sichuan Province Chengdu China; ^7^ Radiology Department Stanford University Stanford California USA; ^8^ Dali Bai Autonomous Prefecture Chinese Medicine Hospital Dali China

**Keywords:** acupuncture, celecoxib, fMRI, knee osteoarthritis, periaqueductal gray, placebo, resting‐state functional connectivity

## Abstract

**Background:**

The analgesic effect of acupuncture is widely recognized, but the mechanical characteristics of acupuncture for pain relief, compared to non‐steroidal anti‐inflammatory (NSAIDs) and placebo medication, remain unknown.

**Aims:**

To compare the modulation effects of acupuncture treatment with NSAIDs and placebo medication on descending pain modulation system (DPMS) in knee osteoarthritis (KOA) patients.

**Methods:**

This study recruited 180 KOA patients with knee pain and 41 healthy controls (HCs). Individuals with KOA knee pain were divided randomly into groups of verum acupuncture (VA), sham acupuncture (SA), celecoxib (SC), placebo (PB), and waiting list (WT), with 36 patients in each group. VA and SA groups included ten sessions of puncturing acupoints or puncturing non‐acupoints acupuncture treatment for two successive weeks. Celecoxib capsules were continuously given orally to patients in the SC group at a dosage of 200 mg daily for 2 weeks. In the PB group, patients received a placebo capsule once a day for 2 weeks at the same dosage as celecoxib capsules. In the WL group, patients did not receive any treatment. Patients underwent a resting‐state BOLD‐fMRI scan pre‐ and post‐receiving the therapy, whereas HCs only underwent a baseline scan. Seed (ventrolateral periaqueductal gray, vlPAG, a key node in DPMS) based resting‐state functional connectivity (rs‐FC) was applied in the data analysis.

**Results:**

All groups demonstrated improved knee pain scores relative to the initial state. There was no statistical difference between the VA and SA groups in all clinical outcomes, and vlPAG rs‐FC alterations. KOA knee pain individuals reported higher vlPAG rs‐FC in the bilateral thalamus than HCs. KOA knee pain patients in the acupuncture group (verum + sham, AG) exhibited increased vlPAG rs‐FC with the right dorsolateral prefrontal cortex (DLPFC) and the right angular, which is associated with knee pain improvement. In contrast with the SC and PB group, the AG exhibited significantly increased vlPAG rs‐FC with the right DLPFC and angular. Contrary to the WT group, the AG showed greater vlPAG rs‐FC with the right DLPFC and precuneus.

**Conclusions:**

Acupuncture treatment, celecoxib, and placebo medication have different modulation effects on vlPAG DPMS in KOA knee pain patients. Acupuncture could modulate vlPAG rs‐FC with brain regions associated with cognitive control, attention, and reappraisal for knee pain relief in KOA patients, compared with celecoxib and placebo medication.

## Graphical abstract

Acupuncture treatment, celecoxib, and placebo medication have different modulation effects on ventrolateral periaqueductal gray (vlPAG) descending pain modulation system (DPMS) in knee osteoarthritis (KOA) knee pain patients. Acupuncture could modulate vlPAG resting‐state functional connectivity (rs‐FC) with brain regions associated with cognitive control, attention, and reappraisal for knee pain relief in KOA patients, compared with celecoxib and placebo medication.

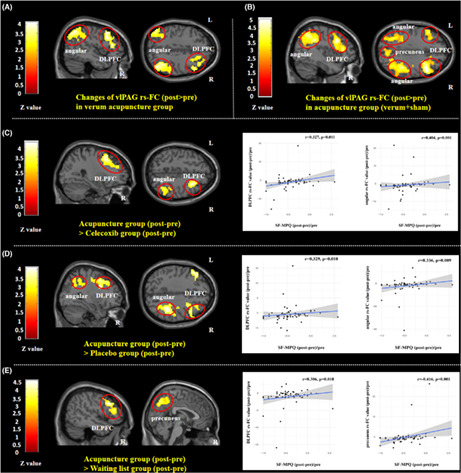

## 1 INTRODUCTION

Knee osteoarthritis (KOA), the most prevalent type of musculoskeletal disease, is characterized by the degeneration of knee cartilage, bone remodeling, and synovitis.^1^, ^2^ KOA is one of the serious global health challenges due to its high prevalence, severe impairment, and enormous societal and financial burden.^3^, ^4^, ^5^, ^6^ Knee pain is the main symptom of KOA that causes patients to seek medical attentions.^7^ Therefore, the treatment guidelines for KOA emphasized pain control as the main therapeutic concept.^8^, ^9^, ^10^, ^11^

The pathophysiology of KOA knee pain is complex. Therefore, understanding the source and mechanism of pain is essential for treatment development. Local indicators of the knee joint and changes in the central nervous system (CNS) play a role in the development of knee pain. Biomechanical variables (including muscle atrophy, overweight, and joint laxity)^1^, ^12^ and biochemical parameters (including inflammatory reactions)^13^, ^14^, ^15^ are examples of local indicators. Functional or structural unbalance in the upstream conduction and downstream pain modulation systems at the spinal cord and brain level are also linked to knee pain in subjects with KOA.^16^, ^17^, ^18^ Treatments for knee pain relief in KOA patients take action through the peripheral, central mechanisms, or both.

The first line recommended medication for treating knee pain caused by KOA in medical practice is NSAIDs medications.^8^, ^19^ The most commonly used NSAID is a selective COX‐2 inhibitor such as celecoxib. Its administration causes minimal hazard gastrointestinal side effects.^20^, ^21^ The peripheral mechanism illustration for celecoxib's analgesia effect is that it reversibly binds to the hydrophilic sac near the activated site of COX‐2. It inhibits the transformation of arachidonic acid to prostaglandin H2, resulting in anti‐inflammatory and pain‐relieving effects.^22^ Previous studies have shown that celecoxib might penetrate the blood–brain barrier (BBB)^23^, ^24^, ^25^ to exert an potential analgesic effect through descending pain modulation system (DPMS).^26^, ^27^, ^28^, ^29^, ^30^ However, side effects and non‐responders come with NSAIDs; thus, there is a growing interest in finding alternative treatments. Acupuncture, an ancient treatment technique of traditional Chinese medicine, has shown its safety and efficacy for KOA knee pain.^19^, ^31^, ^32^, ^33^ Acupuncture analgesia efficacy takes action as a compound therapeutic method through multi‐levels and multi‐factors, of which psychological factor is an important component. Acupuncture has local anti‐inflammatory and analgesia effects.^34^, ^35^, ^36^, ^37^ Beyond that, acupuncture could also modulate the CNS for pain relief. For instance, substantial evidence has implicated that acupuncture could relieve pain by activating DPMS^38^, ^39^, ^40^ and modulating lateral pain pathways (including somatosensory and posterior insula^41^) and medial pain pathways (including dorsal anterior cingulate and anterior insula^41^). However, the characteristic of acupuncture analgesia mechanism on DPMS, especially compared with other treatments, remains largely unknown.

Assessing the physical functioning of DPMS in a non‐invasive manner continues to be difficult. The resting‐state functional magnetic resonance imaging (fMRI) technique allows for the identification of correlations between remote brain regions (resting‐state functional connectivity, rs‐FC) through their highly correlated low‐frequency spontaneous fluctuations non‐invasively.^42^ Recently, many research groups have applied rs‐FC to evaluate the DPMS in humans.^40^, ^43^, ^44^ The periaqueductal gray (PAG) is a mesencephalic brain structure between the third and fourth ventricles.^45^ The PAG has several sub‐regions, of which ventrolateral PAG (vlPAG) is the key node of the DPMS.^46^, ^47^

Furthermore, prior research demonstrated that central sensitization and increased vlPAG activity in osteoarthritis patients are associated with skin irritation in pain areas.^48^, ^49^ Past studies noted that impaired DPMS and treatment modulation, using vlPAG as the seed for rs‐FC, in patients having chronic pain disorders.^40^, ^50^, ^51^, ^52^, ^53^, ^54^, ^55^, ^56^ These results indicated that it is possible to evaluate the DPMS and the modulation effects of treatment in KOA knee pain individuals by utilizing vlPAG rs‐FC in a non‐invasive manner.

In this current work, a comparison of the vlPAG rs‐FC was made between individuals who experienced KOA knee pain and the healthy controls (HCs) matched with them. Furthermore, the mechanism by which the vlPAG rs‐FC could be modulated by different longitudinal interventions, including verum acupuncture, sham acupuncture, celecoxib, placebo medication, and a waiting list, was investigated. Additionally, the possible different vlPAG rs‐FC modulation effects between verum acupuncture and sham acupuncture/celecoxib treatment/placebo medication/waiting list control were compared. We hypothesized that different interventions have different modulation effects on vlPAG DPMS in KOA knee pain patients, and acupuncture could modulate the pain perception by DPMS, allowing psychological factors such as cognitive control, attention, and reappraisal to influence the pain experiences. It is hoped that this study could enhance the understanding of the mechanical characteristics of acupuncture for KOA pain relief compared with NSAIDs and placebo medication.

## 2 METHODS

In this study, the clinical and fMRI datasets of KOA knee pain patients were collected from two simultaneous trials.^57^, ^58^ Notably, all study operations of the two trials were performed under the same site, time period and procedures. The protocols of both research projects were submitted to the Chinese Clinical Trial Registry with the identifiers (ChiCTR‐IOR‐17012364, ChiCTR‐17012365), and they were reviewed by the Sichuan Regional Ethics Review Committee on Traditional Chinese Medicine (ethical approval number: 2016KL‐017). The participants were recruited through the outpatient department of the Hospital of Chengdu University of Traditional Chinese Medicine (TCM), the Third Affiliated Hospital of Chengdu University of TCM, the Sichuan Province Orthopedic Hospital, local posters, the internet, and leaflets. The process of recruitment began in October 2017 and continued until September 2021.

### 2.1 Participants

Knee osteoarthritis knee pain patients and HCs were involved in this study. The diagnostic criteria for KOA were derived from the American College of Rheumatology (ACR) criteria and published in their adjusted form in 1991.^59^ The inclusion criteria were that each patient: (1) was aged 40–60 years and was right‐handed, (2) had continual knee pain throughout the last 3 months, (3) had a mean knee pain score according to the Visual Analog Scale (VAS) ≥3 (range between 0 and 10), (4) had a Kellgren‐Lawrence knee joint radiological degree between 0 and 2, according to the scale,^60^ (5) received no acupuncture treatment or pain killer medicine in the past 3 months and 1 month, respectively, and (6) had signed the written agreement. Individuals met the exclusion criteria if they: (1) were pregnant or lactating women, (2) were alcohol or drug abusers, (3) exhibited mental illness, (4) had severe organic diseases, (5) had rheumatoid arthritis, (6) had any other disorders that caused persistent pain, including a history of head injury that resulted in unconsciousness, (7) had a history of allergies to celecoxib, (8) had contraindications to acupuncture or MRI scan (e.g., claustrophobia), or (9) were currently participating in other trials.

The matched HCs were enrolled. The inclusion criteria were that all HCs: (1) were aged from 40–60 years and were right‐handed, (2) were free from any pain conditions, (3) had no history of mental or CNS illness, (4) had no history of any systemic illness, like diabetes or hypertension, and (5) provided written permissions to conduct the study. HCs met the exclusion criteria if they: (1) were women who were either pregnant or nursing, (2) were alcohol or drug abusers, or (3) had contraindications to MRI scan.

### 2.2 Study design

In this trial, the patients experiencing knee pain due to KOA were observed for 4 weeks, of which the first 2 weeks of run‐in period checked whether KOA patients were eligible and still willing to participate. There were five patient groups and an HCs group, with 36 participants in each group. The patients were randomly assigned to verum acupuncture (VA), sham acupuncture (SA), celecoxib (SC), placebo (PB), and waiting list (WL) control groups. The celecoxib and placebo groups were masked (double‐blinded) to treatment, and the subjects of verum acupuncture and sham acupuncture were kept single‐blinded. Two MRI scans were done on KOA knee pain patients before and after treatments, whereas HCs received one scan.

### 2.3 Intervention

Ten sessions of acupuncture stimulating medication were given to the VA and SA over two successive weeks, each session lasting for 30 min. In the SC group, celecoxib capsules (with an approval number of J20030098 and produced by Pfizer Pharmaceutical Co. Ltd. in Beijing, China) were orally given to patients for 2 weeks of 200 mg. In the PB group, patients received a placebo capsule once a day for 2 weeks at the same dosage as celecoxib capsules. In the WL group, patients did not receive any treatment, but they were informed that after 2 weeks, they would receive ten sessions of acupuncture or 2 weeks of celecoxib for free.

**FIGURE 1:**
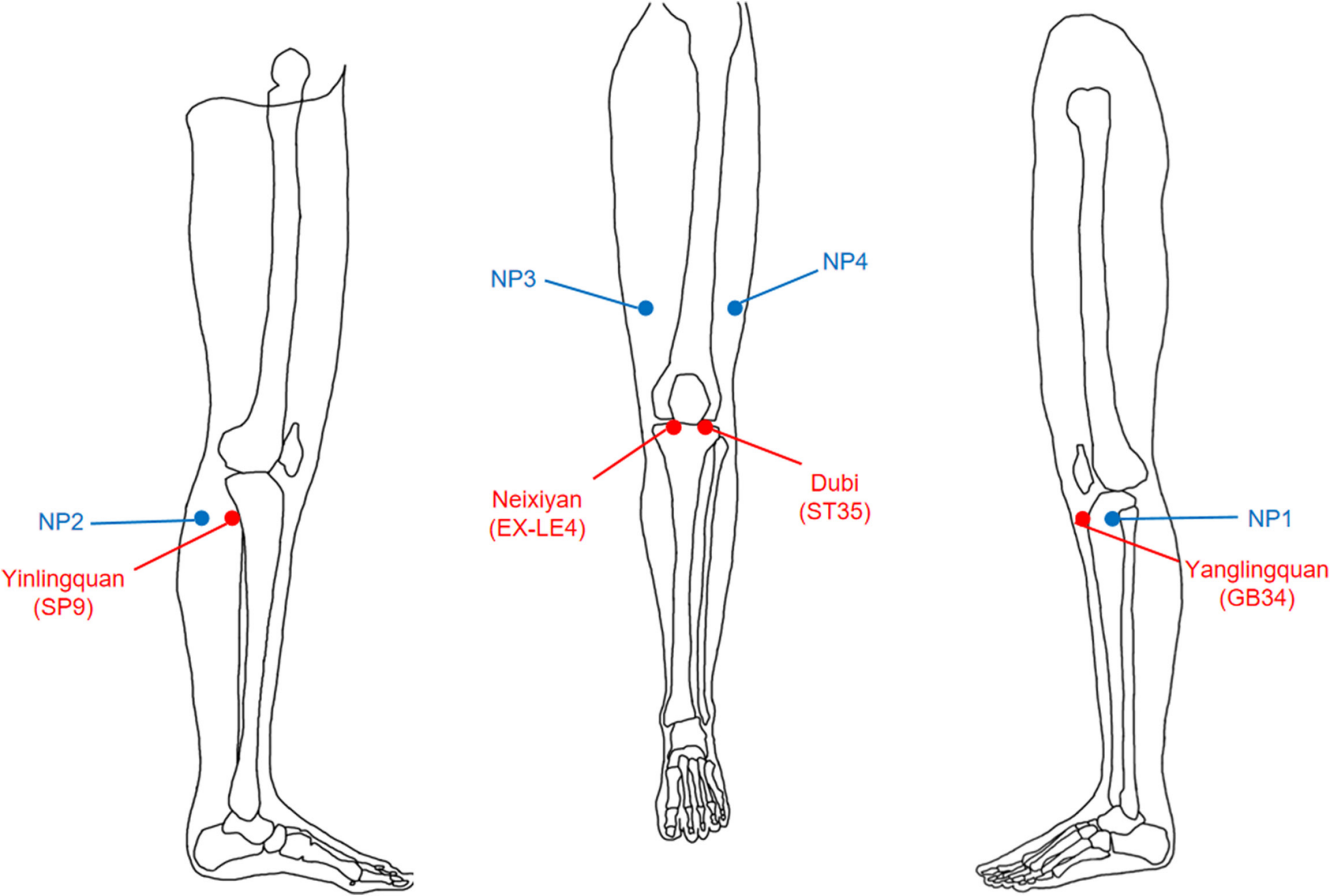
Acupoints and non‐acupoints locations. EX‐LE, Extra points‐lower extremities; GB, Gallbladder meridian of foot shaoyang; NAP, non‐acupoint; SP, Spleen meridian of foot taiyin; ST, Stomach meridian of foot yangming.

The details of acupoint and non‐acupoint selections in the VA and SA group can be seen in this previously published protocol.^57^ VA acupoints were Yanglingquan (GB34), Yinlingquan (SP9), Dubi (ST35), and Neixiyan (EX‐LE4). SA acupoints contained non‐acupoint 1 (NAP1), NAP2, NAP3, and NAP4 (Figure 1). The acupoints and non‐acupoints used in this study were adjacent and located in the knee area.

Two trained and licensed acupuncturists conducted all acupuncture treatments. All acupoints and non‐acupoints on both sides of the body were pierced using small needles made of disposable stainless‐steel measuring 0.25 mm to 40 mm in length (Hwato, China). When inserting the needles, the penetrating depth was kept between 0.5 cun and 1.5 cun. The locations in the VA and SA groups were gently manipulated to induce a specific acupuncture sensation (deqi).

All patients refrained from taking their usual medicines intended to treat KOA during the trial. Ibuprofen, in the form of sustained‐release capsules containing 300 mg, was authorized for use as the rescue therapy in cases of severe pain.

### 2.4 Outcome measurements

This research focused on the vlPAG rs‐FC as its primary endpoint. The clinical assessments were: (1) visual analog scale (VAS), with 0 signifying no pain and 10 signifying the worst pain imaginable,^61^ (2) short‐form McGill pain questionnaire (SF‐MPQ), with the sensory and affective components of pain,^62^ and (3) Western Ontario and McMaster Universities Osteoarthritis Index (WOMAC) with three domains: pain, rigidity, and body activity.^63^ Furthermore, the 12‐item short‐form health survey (SF‐12) was utilized to examine the quality of life of KOA participants related to physical and mental health.^64^ For the KOA knee pain participants, all outcome assessments were taken at the trial baseline after 2 weeks of treatment, while for the HCs, only initial data were retrieved. We recorded the knee pain VAS value of all KOA patients on pre‐ and post‐treatment scans. Patients in the VA and SA groups were requested to complete the Chinese version of the modified Massachusetts General Hospital Acupuncture Sensation Scale (C‐MASS) survey to investigate the possible effect of acupuncture sensation (deqi) on the effectiveness of acupuncture.^65^ Besides, they were also requested to complete the acupuncture expectancy scale (AES) to explore the potential effect of expectation on outcomes.^66^

### 2.5 MRI data acquisition

Sessions of fMRI scanning were conducted at the University of Electronic Science and Technology of China's MRI Center. FMRI brain images were acquired on a 3.0 Tesla system (GE Discovery MR750, GE Healthcare) with an 8‐channel head coil. Before the functional run, T1 MRI images were acquired in three dimensions (TR = 6.008 ms, TE = 1.7 ms, data matrix 256 × 256, the field of view 256 × 256 mm^2^, voxel size 1 × 1 × 1 mm^3^ and scanning duration = 4 min). BOLD resting state functional pictures were acquired using echo‐planar imaging (TR = 2000 ms, TE = 30 ms, flip angle = 90 degrees, slice thickness = 5 mm, acquisition matrix = 64 × 64, voxel size = 2.6 × 2.6 × 2.6 mm^3^, 31 axial slices, total values = 205 and scanning duration = 6:50 min). During the resting state fMRI scan, all subjects were asked to remain conscious, hold their heads steady, close their eyes, and plug their ears.

### 2.6 Patients safety

Adverse events caused by acupuncture and celecoxib, such as pain, bleeding, fainting, gastrointestinal reaction, or other severe events, were processed immediately and recorded in details in the case report form.

### 2.7 Data analysis

#### 2.7.1 Clinical data analysis

All statistical evaluations were performed using SPSS 25.0 (SPSS Inc., Chicago, IL, USA). Normality distribution of the data was assessed, using the visual inspection of histograms and Shapiro–Wilk test. Data that did not follow a normal/Gaussian distribution were analyzed by using non‐parametric test. We used the following statistical statistical tests: chi‐squared test, Mann–Whitney *U* test, and Kruska‐Wallis *H* test. Bonferroni's correction was used to account for multiple comparisons. Statistical analysis was conducted using a 2‐tailed test, and the significance level was set to 5%. Quantitative statistics were presented as medians (lower quartiles; upper quartiles). Frequency was represented as a percentage (%).

#### 2.7.2 vlPAG seed‐based functional connectivity analysis

Functional BOLD information was pre‐processed using DPARSFA (Data Processing Assistant for Resting‐State fMRI, Advanced)^67^ by depending on SPM12 (http://www.fil.ion.ucl.ac.uk/spm) and MATLAB R2013b (Math Works, Inc., Natick, MA, USA). The initial ten‐time points were omitted during pre‐processing for signal stability and subject adaptability. The fMRI images were corrected for slice timing, head motion, segmented, and six head‐movement variables. The white matter signal and cerebrospinal fluid (CSF) signal were regressed. The functional volumes were normalized to the Montreal Neurological Institute (MNI) template space by co‐registering functional pictures with high‐resolution T1‐weighted anatomical images. Subjects were excluded if either translation or rotation of the head motion exceeded ±2 mm or ±2° at any angle. To further limit the detrimental impact of head movement, the volumes with FD >0.5 mm, the one time‐point preceding and two‐time points following the ‘poor’ time points, were eliminated from the data.^68^ The pictures were then detrended, bandpass‐filtered between 0.01 Hz and 0.08 Hz, and smoothed with a 6 mm full‐width half‐maximum Gaussian kernel.

A priori vlPAG seed with the following peak coordinates: 4, −26, −14, and a 3 mm radius was utilized. This seed was already utilized in earlier investigations.^40^, ^43^, ^50^, ^51^, ^69^ There are four reasons why this seed was chosen: (1) the study found that an increase in the degree of heat pain resulted in a significant increase in fMRI signal in the same region,^70^ (2) its site in the vlPAG was perceived as essential for opioid antinociception,^71^, ^72^ (3) celecoxib was discovered to cross the blood–brain barrier (BBB) with the maximum rate of flow accumulated in the midbrain (within PAG),^23^, ^24^, ^25^ and (4) prior research declared that the seed was functionally linked to descending modulatory pain sites at the resting status in healthy control subjects, and a significant variation was observed between healthy subjects and chronic pain subjects.^40^, ^51^, ^56^ Due to the proximity of the vlPAG seed region to a ventricle with a high pulsatile impact, a seed from a neighboring ventricular aqueduct (peak coordinates: 0, −34, −12, with a 3 mm radius) and seeds from the fourth ventricle (peak coordinates: ±4, 8, 12)^40^ were selected as controls.

From the vlPAG seed, the averaged time course was derived, and a voxel‐wise correlation analysis was performed. By applying the regression coefficient between all brain voxels and the time sequence of each seed, contrast pictures for each individual were created. Furthermore, the correlation coefficient map's normality was enhanced by applying Fisher's r‐to‐z transform. The baseline vlPAG rs‐FC of KOA knee pain patients and healthy controls were compared by employing a two‐sample *t*‐test. Pre‐ and post‐treatment values for each group were compared using paired *t*‐tests. Next, factorial design modules were utilized to assess alterations in rs‐FC (post‐treatment minus pre‐treatment) between the verum acupuncture and sham acupuncture groups, acupuncture (verum + sham) and celecoxib groups, acupuncture and placebo medication groups, as well as acupuncture and waiting list groups. Covariables included gender, age, and body mass index (BMI) at baseline. As previous studies mentioned, variables that were percentage transformed could mitigate potential scaling problems.^73^ The association between clinical outcomes and vlPAG rs‐FC was examined by Spearman correlation analysis on the percentage variations (post‐treatment minus pre‐treatment/pre‐treatment) in rs‐FC before and after treatment in the acupuncture group and the corresponding percentage changes in SF‐MPQ. The SPM12 (http://www.fil.ion.ucl.ac.uk/spm/software/spm12) was used to analyze functional connectivity in the rest status. All analyses utilized a threshold of voxelwise *p* < 0.005 (uncorrected) and cluster‐level *p* < 0.05 (family‐wise error correction, FWE).

## 3 RESULTS

**FIGURE 2:**
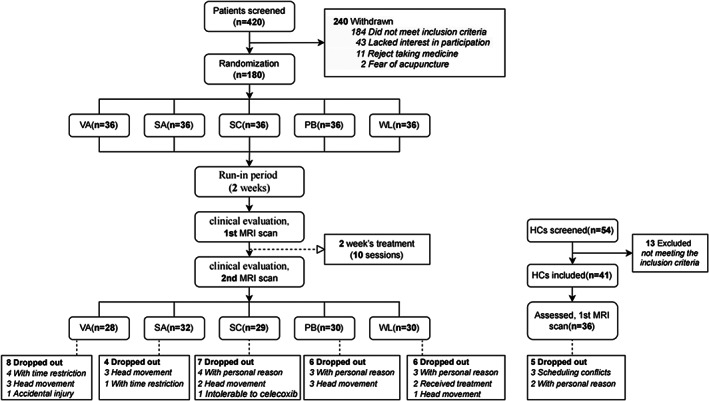
Study flow chart. HCs, healthy controls; PB, placebo group; SA, sham acupuncture group; SC, celecoxib group; VA, verum acupuncture group; WL, waiting list group.

Knee osteoarthritis knee pain patients 180 were recruited, and 31 patients did not complete the study or had excessive head movement. This trial included 41 HCs of the matched age and gender; however, five HCs could not enroll in the MRI scan because of schedule issues. Therefore, 149 KOA knee pain patients and 36 matched HCs were involved in the final analyses (Figure 2).

### 3.1 Baseline characteristics

There was no statistically significant variation between HCs and KOA knee pain subjects following gender, age, or BMI (*p* > 0.05). The VA, SA, SC, PB, and WL groups showed no significant variation in terms of gender, age, BMI, disease duration, VAS, SF‐MPQ, WOMAC, and SF‐12 (*p* > 0.05) (Table 1). Besides, there was no significant difference in knee pain intensity on fMRI scan in KOA patients among all groups (*p* > 0.05) (Table S1).

**TABLE 1:** Baseline characteristics of KOA knee pain patients in different treatment groups and healthy controls.

Characteristics	VA, *n* = 28	SA, *n* = 32	SC, *n* = 29	PB, *n* = 30	WL, *n* = 30	*p* value*	HCs, *n* = 36	KOA, *n* = 149	*p* value**
Female *n* (%)	22 (78.6%)	27 (84.4%)	17 (58.6%)	24 (80.0%)	24 (80.0%)	0.150	23 (63.9%)	114 (76.5%)	0.121
Age (years) M (P_25_, P_75_)	49.00 (45.25, 55.00)	53.50 (48.25, 56.00)	53.00 (50.00, 55.00)	51.50 (49.75, 56.00)	50.00 (47.00, 53.00)	0.295	51.00 (48.00, 54.75)	52.00 (48.00, 55.00)	0.565
BMI (kg/m^2^) M (P_25_, P_75_)	21.82 (20.36, 26.39)	24.10 (22.52, 25.68)	24.03 (22.82, 25.88)	23.34 (21.81, 24.79)	23.94 (21.09, 26.43)	0.103	23.69 (22.06, 26.38)	23.72 (21.49, 25.39)	0.720
Duration (months) M (P_25_, P_75_)	36.00 (13.25, 42.00)	36.00 (19.50, 60.00)	24.00 (12.00, 39.00)	33.00 (16.50, 72.00)	15.00 (6.00, 36.00)	0.118	–	–	–
VAS M (P_25_, P_75_)	4.00 (3.00, 5.00)	4.00 (3.00, 5.00)	4.00 (3.00, 6.00)	4.00 (3.00, 5.00)	3.00 (3.00, 5.00)	0.225	–	–	–
FS‐MPQ M (P_25_, P_75_)	9.25 (7.00, 12.75)	8.00 (7.00, 10.00)	9.00 (8.00, 12.50)	8.00 (6.00, 10.25)	7.00 (6.00, 11.00)	0.068	–	–	–
WOMAC scores M (P_25_, P_75_)	27.00 (13.00, 47.50)	29.50 (16.00, 43.25)	36.00 (23.00, 44.50)	23.50 (13.00, 40.75)	24.00 (18.75, 31.00)	0.415	–	–	–
SF‐12 score M (P_25_, P_75_)	35.50 (30.25, 47.50)	35.00 (32.00, 37.00)	36.00 (32.00, 39.00)	37.50 (33.50, 39.25)	37.00 (36.00, 38.00)	0.116	–	–	–

Abbreviations: BMI, body mass index; HCs, healthy controls; KOA, knee osteoarthritis; MPQ, McGill pain questionnaire; PB, placebo group; SA, sham acupuncture group; SC, celecoxib group; SF‐12, 12‐item short‐form health survey; VA, verum acupuncture group; VAS, visual analog scale; WL, waiting list group; WOMAC, Western Ontario and McMaster Universities Arthritis Index.

* Kruskal‐Wallis *H* test was applied for comparing VA, SA, SC, PB, and WL groups.

** Mann–Whitney *U* test was applied for comparing HCs and KOA groups. A *p* value <0.05 was considered statistically significant.

### 3.2 Clinical outcomes

All groups demonstrated improvement in VAS, SF‐MPQ, WOMAC, and SF‐12 scores relative to the initial group (*p* < 0.05; Table 2). Mann–Whitney *U* test showed the changes in VAS, SF‐MPQ, and WOMAC were statistically different between the SC and PB groups (Table 3). Nevertheless, there was no statistical difference between the VA and SA groups in all outcomes (VAS, SF‐MPQ, WOMAC, and SF‐12), which was similar to the previous research on RCTs and meta‐analyses, implying that the particular impact of perforating acupoints over perforating non‐acupoints for knee pain is none or only a small to moderate.^33^, ^74^, ^75^, ^76^, ^77^, ^78^ Besides, AES scores did not differ significantly between VA and SA groups for expectations of acupuncture (*p* = 0.777; Table S2). The average C‐MASS ratings for all ten descriptors showed no statistical difference in acupuncture sensations between VA and SA groups (*p* = 0.080; Table S2). Based on the above, the VA and SA groups were combined into a single acupuncture group (AG) to study the overall acupuncture effect. Mann–Whitney *U* test with Bonferroni correction was applied to compare AG and SC, AG and PB, and AG and WL groups, respectively. Results showed no statistically significant differences between the AG and SC groups, which was similar to the previous RCT, suggesting that acupuncture and NSAIDs had a similar analgesic impact on controlling pain.^19^ There were statistical differences between the AG and PB groups, and the AG and WL groups, in the changes of VAS and SF‐MPQ but not in WOMAC and SF‐12 (Table 3).

**TABLE 2:** Clinical variables at the baseline and the end of the study in different groups.

Outcome measures	VA, *n* = 28	SA, *n* = 32	SC, *n* = 29	PB, *n* = 30	WL, *n* = 30	AG, *n* = 60
VAS M (P_25_, P_75_)						
Baseline	4.00 (3.00, 5.00)	4.00 (3.00, 5.00)	4.00 (3.00, 6.00)	4.00 (3.00, 5.00)	3.00 (3.00, 5.00)	4.00 (3.00, 5.00)
End of treatment	2.50 (1.00, 3.00)	3.00 (2.00, 3.00)	3.00 (1.50, 3.00)	3.00 (3.00, 4.00)	3.00 (2.00, 3.25)	3.00 (2.00, 3.00)
*p* value	<0.001	<0.001	<0.001	0.002	0.001	<0.001
SF‐MPQ M (P_25_, P_75_)						
Baseline	9.25 (7.00, 12.75)	8.00 (7.00, 10.00)	9.00 (8.00, 12.50)	8.00 (6.00, 10.25)	7.00 (6.00, 11.00)	8.00 (7.00, 12.00)
End of treatment	4.50 (2.00, 7.75)	6.00 (4.00, 7.75)	5.00 (4.00, 7.00)	6.00 (5.00, 8.00)	6.00 (4.75, 7.00)	5.00 (3.00, 7.75)
*p* value	<0.001	<0.001	<0.001	<0.001	<0.001	<0.001
WOMAC scores M (P_25_, P_75_)						
Baseline	27.00 (13.00, 47.50)	29.50 (16.00, 43.25)	36.00 (23.00, 44.50)	23.50 (13.00, 40.75)	24.00 (18.75, 31.00)	27.50 (14.25, 44.00)
End of treatment	11.50 (2.50, 23.50)	14.50 (6.50, 24.25)	15.00 (8.00, 28.50)	17.50 (8.75, 28.00)	17.50 (12.00, 21.50)	13.50 (5.00, 23.50)
*p* value	<0.001	<0.001	<0.001	<0.001	<0.001	<0.001
SF‐12 score M (P_25_, P_75_)						
Baseline	35.50 (30.25, 37.50)	35.00 (32.00, 37.00)	36.00 (32.00, 39.00)	37.50 (33.50, 39.25)	37.00 (36.00, 38.00)	35.00 (31.25, 37.00)
End of treatment	36.50 (32.00, 41.00)	37.00 (35.25, 40.75)	39.00 (35.50, 40.50)	38.00 (36.00, 40.00)	38.00 (36.75, 39.00)	37.00 (35.00, 41.00)
*p* value	0.010	0.001	0.002	0.006	0.043	<0.001

*Note*: The Wilcoxon signed‐rank test was applied for comparison in each group. A *p* value <0.05 was considered statistically significant.

Abbreviations: AG, acupuncture group (VA + SA); PB, placebo group; SA, sham acupuncture group; SC, celecoxib group; SF‐12, 12‐item short‐form health survey; SF‐MPQ, short‐form McGill pain questionnaire; VA, verum acupuncture; VAS, visual analog scale; WL, waiting list group; WOMAC, Western Ontario and McMaster Universities Arthritis Index.

**TABLE 3:** Comparisons of the therapeutic effect among different groups.

Outcome measures	VA, *n* = 28	SA, *n* = 32	*p* value*	SC, *n* = 29	PB, *n* = 30	*p* value*	AG, *n* = 60	SC, *n* = 29	*p* value**	AG, *n* = 60	PB, *n* = 30	*p* value**	AG, *n* = 60	WL, *n* = 30	*p* value**
VAS M (P_25_, P_75_)															
End of treatment	2.50 (1.00, 3.00)	3.00 (2.00, 3.00)	0.465	3.00 (1.50, 3.00)	3.00 (3.00, 4.00)	0.013	3.00 (2.00, 3.00)	3.00 (1.50, 3.00)	0.870	3.00 (2.00, 3.00)	3.00 (3.00, 4.00)	0.015	3.00 (2.00, 3.00)	3.00 (2.00, 3.25)	0.330
End ‐ baseline	−2.00 (−3.00, −1.00)	−1.25 (−2.75, −0.63)	0.181	−2.00 (−3.00, −1.00)	−1.00 (−2.00, 0.00)	0.002	−2.00 (−3.00, −1.00)	−2.00 (−3.00, −1.00)	0.609	−2.00 (−3.00, −1.00)	−1.00 (−2.00, 0.00)	0.001	−2.00 (−3.00, −1.00)	−1.00 (−2.00, 0.00)	0.015
SF‐MPQ M (P_25_, P_75_)															
End of treatment	3.00 (1.50, 3.00)	3.00 (3.00, 4.00)	0.388	5.00 (4.00, 7.00)	6.00 (5.00, 8.00)	0.333	5.00 (3.00, 7.75)	5.00 (4.00, 7.00)	0.316	5.00 (3.00, 8.00)	6.00 (5.00, 8.00)	0.073	5.00 (3.00, 8.00)	6.00 (4.75, 7.00)	0.342
End ‐ baseline	−4.00 (−7.00, −3.00)	−4.00 (−5.00, −2.00)	0.227	−4.00 (−6.00, −2.00)	−2.00 (−3.63, 0.00)	0.002	−4.00 (−7.00, −2.13)	−4.00 (−6.00, −2.00)	0.961	−4.00 (−7.00, −2.13)	−2.00 (−3.63, 0.00)	<0.001	−4.00 (−7.00, −2.13)	−2.00 (−4.25, −1.00)	0.002
WOMAC M (P25, P75)															
End of treatment	14.00 (4.00, 31.00)	14.50 (6.50, 24.25)	0.441	15.00 (8.00, 28.50)	17.50 (8.75, 17.50)	0.739	13.50 (5.00, 23.50)	15.00 (8.00, 28.50)	0.283	11.50 (2.50, 23.50)	17.50 (8.75, 17.50)	0.149	11.50 (2.50, 23.50)	16.00 (11.25, 21.50)	0.191
End ‐ baseline	−13.50 (−20.50, −7.00)	−13.00 (−24.25, −3.50)	0.876	−16.00 (−27.00, −8.50)	−5.00 (−13.25, −2.00)	0.004	−13.00 (−21.75, −6.00)	−16.00 (−27.00, −8.50)	0.346	−13.00 (−21.75, −6.00)	−5.00 (−13.25, −2.00)	0.023	−13.00 (−21.75, −6.00)	−6.50 (−17.25, −1.00)	0.047
SF‐12 score M (P_25_, P_75_)															
End of treatment	36.00 (32.00, 41.00)	37.00 (35.25, 40.75)	0.699	39.00 (35.50, 40.50)	38.00 (36.00, 40.00)	0.516	37.00 (35.00, 41.00)	39.00 (35.50, 40.50)	0.533	37.00 (34.00, 41.00)	38.00 (36.00, 40.00)	0.767	37.00 (34.00, 41.00)	37.50 (36.75, 38.25)	0.928
End ‐ baseline	2.00 (−0.75, 5.00)	2.00 (0.25, 5.00)	0.806	1.00 (0.00, 4.00)	1.50 (0.00, 2.50)	0.566	1.00 (0.00, 4.00)	1.00 (0.00, 4.00)	0.518	1.00 (0.00, 4.00)	1.50 (0.00, 2.50)	0.192	1.00 (0.00, 4.00)	0.50 (−0.25, 2.25)	0.024

Abbreviations: AG, acupuncture group (VA + SA); PB, placebo group; SA, sham acupuncture; SC, celecoxib group; SF‐12, 12‐item short‐form health survey; SF‐MPQ, short‐form McGill pain questionnaire; VA, verum acupuncture; VAS, visual analog scale; WL, waiting list group; WOMAC, Western Ontario and McMaster Universities Arthritis Index.

* Mann–Whitney *U* test was applied to compare VA and SA, SC, and PB groups. A *p* value <0.05 was considered statistically significant.

** Mann–Whitney *U* test with Bonferroni correction was applied to compare AG and SC groups, AG and PB groups, and AG and WL groups, respectively. A *p* value <0.017 was considered statistically significant.

### 3.3 vlPAG rs‐FC results

#### 3.3.1 KOA vs. HCs

**FIGURE 3:**
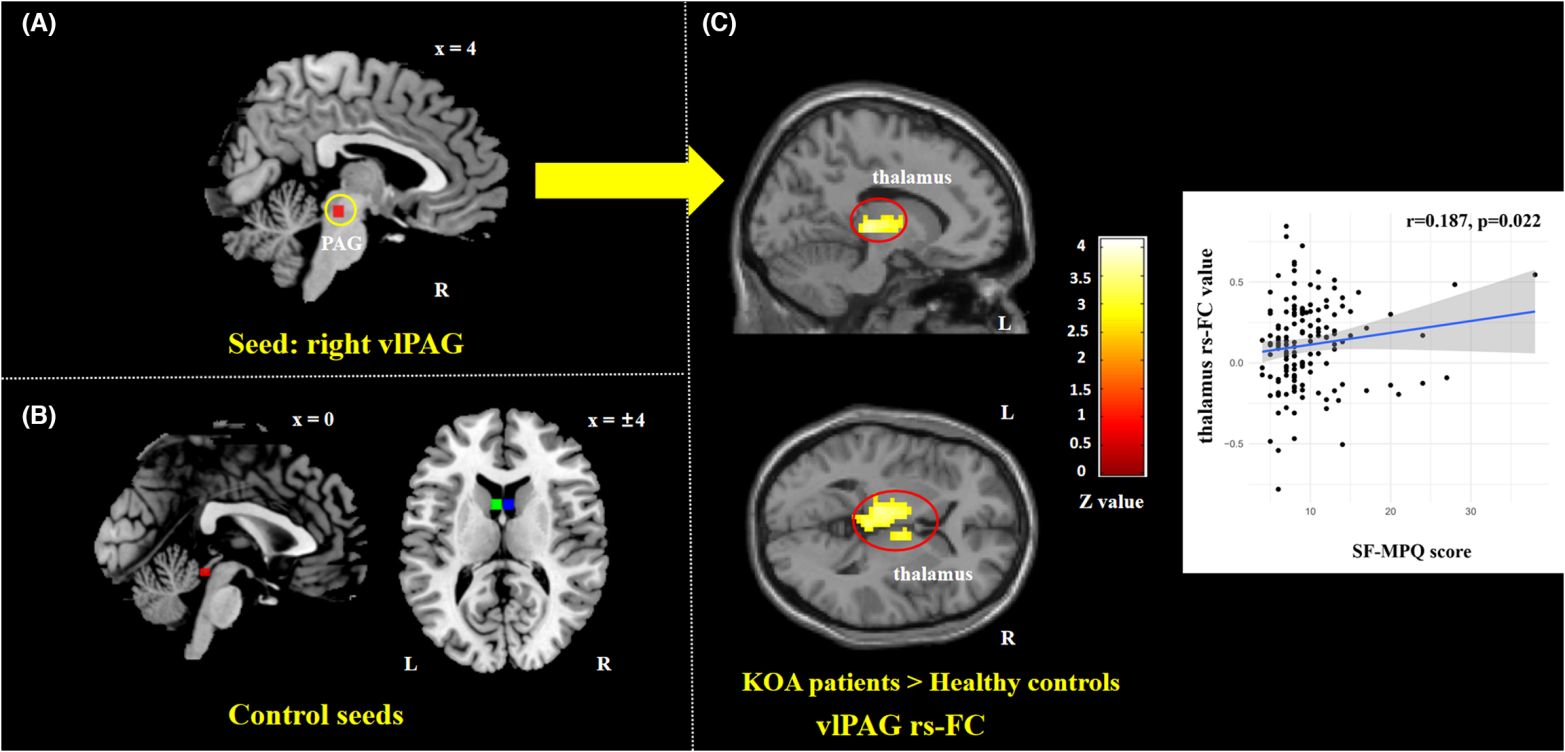
Comparisons of vlPAG seed and control seeds, and vlPAG rs‐FC between KOA and HCs. (A) The right vlPAG (*x* = 4, *y* = −26, *z* = −14, with 3 mm radius) seed. (B) Given that the vlPAG seed region sits adjacent to the ventricle with a significant pulsatile effect, three seeds from the ventricular aqueduct nearby the vlPAG seed were chosen as control seeds. Red, a seed from a ventricular aqueduct nearby (peak coordinate: 0, −34, −12, with 3 mm radius); Green, a seed from the left fourth ventricle (peak coordinate: −4, 8, 12, with 3 mm radius); Blue, a seed from the right fourth ventricle (peak coordinate: 4, 8, 12, with 3 mm radius). (C) vlPAG seed‐based functional connectivity between KOA patients and HCs. Spearman correlation analyses between the bilateral thalamus rs‐FC levels and FS‐MPQ. HCs, healthy controls; KOA, knee osteoarthritis; L, left; R, right; rs‐FC, resting‐state functional connectivity; vlPAG, ventrolateral periaqueductal gray.

Compared with HCs, KOA knee pain patients showed increased vlPAG rs‐FC with bilateral thalamus (Figure 3C, Table 4). Spearman correlation analyses demonstrated that rs‐FC levels of the bilateral thalamus had a small but significant positive correlation with SF‐MPQ (*r* = 0.187, *p* = 0.022) (Figure 3C).

**TABLE 4:** The vlPAG rs‐FC in KOA knee pain patients and the modulation effects in different groups.

A. vlPAG rs‐FC difference between KOA and HCs							B. vlPAG rs‐FC difference between verum acupuncture group and sham acupuncture group							C. vlPAG rs‐FC changes in acupuncture group (verum + sham)						
Contrast	Voxels	Brain Region	MNI (*x*, *y*, *z*)			*Z*	Contrast	Voxels	Brain Region	MNI (*x*, *y*, *z*)			*Z*	Contrast	Voxels	Brain region	MNI (*x*, *y*, *z*)			*Z*
KOA > HCs	346	L thalamus	−12	−24	3	3.60	VA > SA (post‐pre)		None					Post > Pre	1786	B DLPFC	42	15	33	4.98
		R thalamus	6	−21	15	3.05									1945	B angular	39	−51	45	4.64
															248	B precuneus	3	−69	39	3.95
KOA < HCs		None					VA < SA (post‐pre)		None					Post < Pre		None				

D. vlPAG rs‐FC changes in celecoxib group							E. vlPAG rs‐FC changes in placebo group							F. vlPAG rs‐FC changes in waiting list group						
Contrast	Voxels	Brain region	MNI (*x*, *y*, *z*)			*Z*	Contrast	Voxels	Brain region	MNI (*x*, *y*, *z*)			*Z*	Contrast	Voxels	Brain region	MNI (*x*, *y*, *z*)			*Z*
Post > Pre		None					Post > Pre		None					Post > Pre		None				
Post < Pre		None					Post < Pre		None					Post < Pre		None				

G. vlPAG rs‐FC difference between acupuncture group and celecoxib group							H. vlPAG rs‐FC difference between acupuncture group and placebo group							I. vlPAG rs‐FC difference between acupuncture group and waiting list group						
Contrast	Voxels	Brain Region	MNI (*x*, *y*, *z*)			*Z*	Contrast	Voxels	Brain region	MNI (*x*, *y*, *z*)			*Z*	Contrast	Voxels	Brain region	MNI (*x*, *y*, *z*)			*Z*
AG > SC (post‐pre)	403	R DLPFC	24	12	54	4.32	AG > PB (post‐pre)	748	B DLPFC	42	15	30	4.10	AG > WL (post‐pre)	753	R DLPFC	51	18	33	4.43
	255	R angular	39	−54	48	3.40		594	R angular	39	−51	45	3.91		276	R precuneus	6	−66	48	3.55
AG < SC (post‐pre)		None					AG < PB (post‐pre)		None					AG < WL (post‐pre)		None				

*Note*: A threshold of a voxel‐wise *p* < 0.005 uncorrected and *p* < 0.05 family‐wise error (FWE) correction at cluster level were applied.

Abbreviations: AG, acupuncture group (verum + sham); B, bilateral; DLPFC, dorsolateral prefrontal cortex; HCs, healthy controls; KOA, knee osteoarthritis; L, left side; R, right; rs‐FC, resting state functional connectivity; SA, sham acupuncture group; SC, celecoxib group; VA, verum acupuncture group; WL, waiting list group.

#### 3.3.2 Modulation effects of different treatments

**FIGURE 4:**
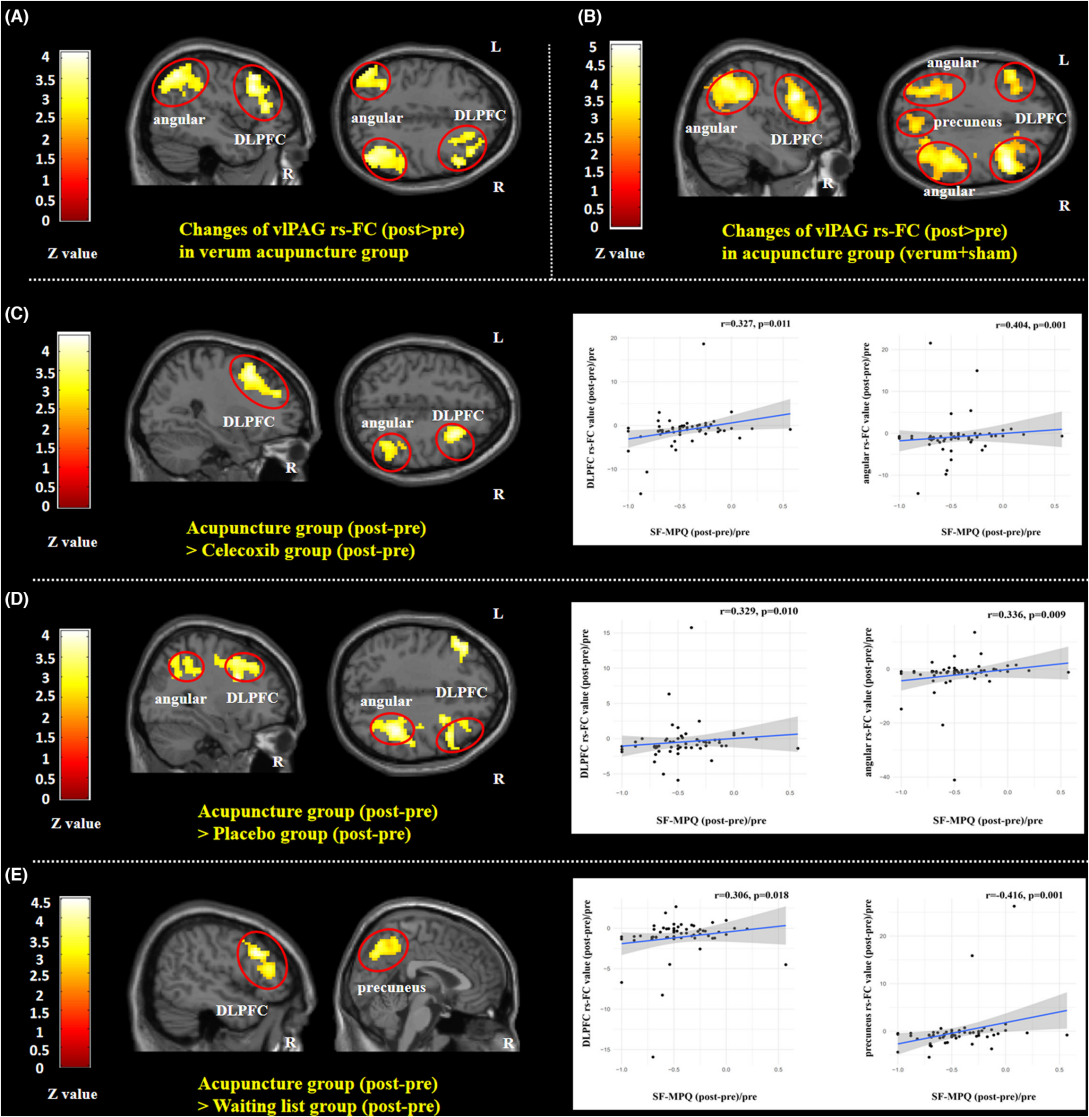
vlPAG rs‐FC alternations in different treatment groups and comparison between acupuncture (verum + sham) and celecoxib, placebo, and waiting list control. (A) Brain regions showed vlPAG rs‐FC increased (post‐ minus pre‐treatment) in the verum acupuncture treatment group. (B) Brain regions showed the vlPAG rs‐FC increased (post‐ minus pre‐treatment) in the acupuncture treatment group (verum + sham). (C–E) Brain regions showed greater vlPAG rs‐FC increase (post‐ minus pre‐treatment) in the acupuncture treatment group (verum + sham) compared to the celecoxib group, placebo group, and waiting list group. Increased rs‐FC between the right vlPAG and right DLPFC/angular/precuneus after acupuncture (verum + sham) treatment was associated with reduced KOA knee pain intensity in patients who received acupuncture treatment. DLPFC, dorsolateral prefrontal cortex; KOA, knee osteoarthritis; L, left side; R, right side; rs‐FC, resting state functional connectivity; SF‐MPQ, short‐form McGill pain questionnaire; vlPAG, ventrolateral periaqueductal gray. *percentage change means post‐treatment minus pre‐treatment/pre‐treatment.

After longitudinal VA treatment, KOA knee pain subjects had increased vlPAG rs‐FC with the right dorsolateral prefrontal cortex (DLPFC), and bilateral angular (Figure 4A, Table 4). Nevertheless, there were no significant alterations in vlPAG rs‐FC in KOA knee pain subjects following longitudinal medication with celecoxib, placebo, or waiting list. Moreover, VA and SA groups in terms of vlPAG rs‐FC alterations showed no significant variation (Table 4).

According to the absence of significant variation in clinical outcomes and vlPAG rs‐FC alterations between VA and SA groups (Table 4), the two groups were combined into a single acupuncture group (AG). After the longitudinal AG treatment, KOA knee pain subjects had increased rs‐FC vlPAG with the bilateral dorsolateral prefrontal cortex (DLPFC), bilateral angular, and bilateral precuneus (Figure 4B, Table 4). More comparisons were made to investigate the potential variation in vlPAG rs‐FC regulation impact between acupuncture and celecoxib, placebo, and waiting list. In contrast with the celecoxib group, the AG exhibited increased vlPAG rs‐FC with the right DLPFC and right angular (Figure 4C, Table 4). Compared with the placebo group, the AG showed greater vlPAG rs‐FC with the right DLPFC and right angular (Figure 4D, Table 4). Contrary to the waiting list group, the AG exhibited significantly increased vlPAG rs‐FC with the right DLPFC and right precuneus (Figure 4E, Table 4). Furthermore, to analyze the correlation between vlPAG rs‐FC alterations and medical factors, the Fisher‐*z* value was obtained for the rs‐FC in the right DLPFC/angular/precuneus site. It was discovered that the proportion alterations of rs‐FC between vlPAG and DLPFC/angular/precuneus were positively correlated with the matched SF‐MPQ percentage alterations in the acupuncture group (Figure 4C–E).

As additional controls, the previously mentioned analysis was performed using the seeds from the ventricular aqueduct near the vlPAG and from the fourth ventricle. No outcome was detected, which additionally validated the outcomes of vlPAG rs‐FC in this investigation.

### 3.4 Medication uses

No patient reported pain medication usage during the 4‐week observation period in this study. However, three patients used plaster on the local knee joint for pain relief (two in the placebo group and one in the celecoxib group).

### 3.5 Patients safety

All 180 patients were monitored for safety and tolerability. One patient in the VA group discontinued the study due to a fractured upper limb unrelated to the treatment. Four patients in SA and four patients in VA groups developed mild bruising around the acupoints/non‐acupoints, all of which were back to normal after 2 weeks of follow‐up. One patient discontinued celecoxib during the intervention period due to intolerable gastric discomfort, and no further gastric discomfort occurred after 2 weeks of follow‐up.

## 4 DISCUSSION

This study explored the vlPAG rs‐FC alternations and the modulation effects of verum, sham acupuncture, celecoxib, placebo, and waiting list treatment in KOA knee pain patients. The clinical and vlPAG rs‐FC results showed no significant difference between VA and SA groups. Acupuncture (VA + SA) and celecoxib groups had similar clinical effects on pain relief in KOA knee pain patients. Furthermore, the acupuncture group showed a significantly increased vlPAG rs‐FC with the right DLPFC and right angular, compared with celecoxib treatment, placebo, and waiting list groups. Interestingly, these vlPAG rs‐FC changes were correlated with a decreasing KOA pain intensity in the acupuncture group. To our knowledge, this is an initial study exploring the mechanical characteristics of acupuncture on DPMS compared with NSAIDs and placebo medications for KOA knee pain relief.

### 4.1 Verum acupuncture versus sham acupuncture

This study found that verum acupuncture (puncturing acupoints) and sham acupuncture (puncturing non‐acupoints) were all equally effective for KOA knee pain relief and had similar effects on vlPAG rs‐FC, which is similar to the findings from previously published literature on RCTs and meta‐analyses. Some research reported no advantage of puncturing acupoints over non‐acupoints for treating knee pain,^74^, ^75^, ^76^ while other studies showed that puncturing acupoints was more effective than puncturing non‐acupoints, but the impact size was only a small to moderate.^33^, ^77^, ^78^ The specificity effect of acupoints for pain relief remains controversial, which needs further investigation.

### 4.2 Acupuncture versus celecoxib

This study found no clinical therapeutic efficacy difference in knee pain relief between acupuncture treatment and celecoxib, which further validated the analgesic effect of acupuncture. Celecoxib is the most widely used NSAIDs for the medication of KOA.^21^ Several researchers also declared that acupuncture was at least as effective as celecoxib in relieving knee pain for KOA patients.^19^, ^79^ Although both celecoxib and acupuncture could relieve knee pain for KOA patients in clinical practice, they have different analgesia mechanisms.

The neuroimaging results showed that KOA knee pain patients had increased vlPAG rs‐FC with bilateral thalamus compared with HCs. Neuroanatomical studies have found that the thalamus has neural fiber connections with PAG through the thalamus‐PAG pathway,^80^, ^81^, ^82^, ^83^ which is part of the pathway in DPMS. This finding indicated that KOA knee pain might have impaired DPMS function. This study also found that the acupuncture (VA + SA) group exhibited a significantly attenuated rs‐FC between the vlPAG and the right DLPFC and right angular, compared to the celecoxib group which were also correlated with suppression in KOA pain severity. These results indicate that celecoxib and acupuncture treatment might have different modulation effects on DPMS in KOA knee pain patients. Previous studies have reported that celecoxib could exert an analgesic effect through peripheral and central mechanisms for many chronic pain conditions.^26^, ^27^, ^28^, ^29^, ^30^

Nevertheless, this study found that celecoxib did not affect vlPAG rs‐FC in KOA patients. It is believed that celecoxib might take action for KOA knee pain relief mainly through the local anti‐inflammatory and analgesic mechanism in the knee joint, with limited involvement of vlPAG DPMS at the supraspinal level. Unlike celecoxib, acupuncture could modulate vlPAG rs‐FC with the right DLPFC and angular for knee pain relief in KOA patients. Research has shown that the DLPFC is directly connected to PAG through DLPFC‐thalamus‐PAG/DLPFC‐ACC‐thalamus‐PAG pathway,^80^, ^81^, ^82^, ^83^, ^84^ a typical DPMS at the supraspinal level. Abundant evidence has indicated the contribution of DLPFC to cognitive control of pain through attention, anticipation, and reappraisal.^47^, ^80^, ^87^, ^88^, ^89^ Angular locates in the posterior part of the inferior parietal lobe, and the angular gyrus has direct connections with DLPFC via the superior longitudinal fasciculus‐second branch (SLF‐II).^90^ The right angular and DLPFC together constitute the two most important hubs of the right frontoparietal network (cognition control network),^91^ which performs a significant part in cognitive and top‐down regulations.^92^ Past studies revealed that expectancy, focus, and revaluations are critical elements of the non‐specific effect of therapies, such as acupuncture.^80^, ^84^, ^93^ These findings implied that acupuncture might alleviate KOA knee pain by modulating brain regions associated with cognitive control, attention, and reappraisal through DPMS. Acupuncture is a more complex and compound intervention compared with celecoxib. Past research has revealed that acupuncture could suppress inflammation and pain factor levels in the knee joint.^34^, ^35^, ^36^, ^37^, ^85^ Besides the peripheral mechanism, this study found that acupuncture could also impact vlPAG DPMS for knee pain relief. However, more in‐depth and comprehensive studies need to be done to find the impact size of acupuncture modulation on DPMS in the overall impact size of acupuncture for KOA knee pain relief.

### 4.3 Acupuncture versus classic placebo medication

This study found that acupuncture treatment had a better therapeutic effect than placebo medication in knee pain relief for KOA patients. The neuroimaging results showed that the AG exhibited increased vlPAG rs‐FC with the bilateral DLPFC and the right angular compared with the PB group. The acupuncture effect has long been considered a magic placebo in western countries. Although several studies indicate that acupuncture is not a placebo,^39^, ^93^, ^94^, ^95^ it is undeniable that the placebo effect played an important role in acupuncture analgesia.^96^ A recent network meta‐analysis showed that intra‐articular and topical placebos were superior to oral placebo in reducing knee pain,^97^ which indicates that the forms of the placebo can significantly affect the efficacy of the placebo. This meta‐analysis study might help to explain why acupuncture, as an invasive treatment, exhibited a better pain relief effect than oral placebo medication for KOA patients. One neural mechanism for the increased placebo effect following invasive procedures may be that the perceived invasiveness of acupuncture leads to the presence of belief‐based rewards engaged the DPMS within the PAG, which directly affects the analgesic effect of acupuncture.^98^

Studies have shown that endogenous opioid and non‐opioid mechanisms (e.g., dopamine and endocannabinoids) may mediate placebo analgesia.^99^, ^100^ A neuroimaging study reported that the placebo response was accompanied by decreased activation of brain regions, including the anterior cingulate cortex, prefrontal cortex, insula, thalamus, amygdala, nucleus accumbens, and PAG,^101^ which are the main brain regions in opioid‐mediated DPMS and dopamine‐mediated reward system.^102^, ^103^ Previous studies have shown that the analgesic effects of acupuncture are mediated, at least in part, through the DPMS.^93^ This study had similar findings; however, no vlPAG rs‐FC alternation in KOA patients treated with placebo capsules was found. It is speculated that placebo medication might also exert analgesic effects through non‐descending pain pathways, such as a reward system, for knee pain relief. Based on the above results, it is believed that there are different mechanisms underlying acupuncture analgesia and expectancy‐evoked placebo analgesia.

This research had the following drawbacks: (1) each group's sample size was relatively small, preventing us from comparing clinical outcome variations across the different groups; (2) the inflammation and pain‐related variables in the knee joint were not observed and investigated, preventing a comprehensive analysis of acupuncture and celecoxib mechanism for KOA knee pain; (3) the course of acupuncture and celecoxib treatment for KOA patients was relatively too short, which might have influenced the clinical and neuroimaging results; (4) there may have been a risk of bias between the two concurrent trials in this study; (5) neither KOA patients in the celecoxib nor placebo groups received nocebo‐related psychological assessments in this study, which might have a potential impact on the clinical and fMRI results in this study; (6) this study only observed DPMS from the perspective of resting‐state functional connectivity in KOA knee pain patients, and structural connectivity as a topic worth studying in the future; and (7) the fMRI data with cardiac‐gating was not obtained^104^ and the respiratory or heart rate records were not noted to account for probable physiological and movement artifacts. In data pre‐processing, however, the motion, CSF, and white matter signals were all regressed away. Notably, the possible physiological/movement impact required no region‐specificity. Additionally, the absence of significant control seeds in the ventricle confirmed the conclusion. Further investigations may be required to compare fMRI data in the absence or presence of cardiac gating and physiological adjustments.

## 5 CONCLUSION

Acupuncture treatment, celecoxib, and placebo medication have different modulation effects on vlPAG DPMS in KOA knee pain patients. Acupuncture could modulate vlPAG rs‐FC with brain regions associated with cognitive control, attention, and reappraisal for knee pain relief in KOA patients, compared with celecoxib and placebo medication.

## AUTHOR CONTRIBUTIONS

ZL and FL are the corresponding authors. JZ and FZ contributed equally to this article. Study protocol and design: FL, ZL and FZ; acquisition of data: SC, XD, XZ, CT, WH, YC, NJ, NS, YZ, XL and SH; analysis and interpretation of data: JZ, ZL, WY and RS; drafting of the manuscript: JZ and FZ; proofread and revise the manuscript: ZL, FL, and MW. All authors reviewed the manuscript.

## FUNDING INFORMATION

This study was supported by funds from the National Natural Science Foundation of China (No.81973958, No.81774400, No.81603708, and No.82225050), Innovation Team and Talents Cultivation Program of National Administration of Traditional Chinese Medicine (No.ZYYCXTD‐D‐202003), and Postdoctoral Science Foundation (No.2017M610593, No.2018T110954, and No.PC2019012).

## CONFLICT OF INTEREST STATEMENT

None of the authors have any conflict of interest.

## Supporting information


Table S1.

Table S2.

## Data Availability

The datasets used and/or analyzed during the current study are available from the corresponding author on reasonable request.
