# Supplementary material for: Modulation effects of different treatments on periaqueductal gray resting state functional connectivity in knee osteoarthritis knee pain patients
Source: CNS Neurosci Ther. 2023 Mar 8;29(7):1965–80. doi: 10.1111/cns.14153 (PMC10324370; doi:10.1111/cns.14153)
Supplement: Supplementary file 1 — Table S1. [file CNS-29-1965-s002.docx]

**Table S1. Comparisons of the KOA patients suffering from knee pain during the fMRI scan in different groups.**

| **Outcome measures** | **VA, n=28** | **SA, n=32** | **SC, n=29** | **PB, n=30** | **WL, n=30** | **P** value****** |
| --- | --- | --- | --- | --- | --- | --- |
| **VAS**  **M (P_25_, P_75_)** |  |  |  |  |  |  |
| 1^st^ MRI scan | 0.00  (0.00, 1.00) | 0.00  (0.00, 0.75) | 0.00  (0.00, 0.00) | 0.00  (0.00, 2.00) | 0.00  (0.00, 0.00) | 0.400 |
| 2^nd^ MRI scan | 0.00  (0.00, 0.00) | 0.00  (0.00, 0.00) | 0.00  (0.00, 0.00) | 0.00  (0.00, 0.00) | 0.00  (0.00, 0.25) | 0.621 |
| *P* value***** | 0.081 | 0.418 | 0.572 | 0.169 | 0.435 |  |

MRI, magnetic resonance imaging; PB, placebo group; SA, sham acupuncture group; SC, [celecoxib](javascript:;) group; VA, verum acupuncture; VAS, visual analog scale; WL, waiting list group; *The Wilcoxon signed-rank test was applied for comparison in each group. **Kruskal-Wallis H test was applied for comparing VA, SA, SC, PB, and WL groups; A *P* value < 0.05 was considered statistically significant.
