# Supplementary material for: Modulation effects of different treatments on periaqueductal gray resting state functional connectivity in knee osteoarthritis knee pain patients
Source: CNS Neurosci Ther. 2023 Mar 8;29(7):1965–80. doi: 10.1111/cns.14153 (PMC10324370; doi:10.1111/cns.14153)
Supplement: Supplementary file 2 — Table S2. [file CNS-29-1965-s001.doc]

**Table S2. Comparisons of the acupuncture sensation and expectation between verum and sham acupuncture groups.**

| **Outcome measures** | **VA, n=28** | **SA**, n=32 | **P value**  ***** |
| --- | --- | --- | --- |
| **AES**  **M (P**_25_, P_75_) |  |  |  |
| baseline | 13.00 | 10.50 | 0.777 |
|  | (8.25, 14.00) | (8.00, 18.00) |  |
| 1. **MASS**   **M (P**_25_, P_75_) |  |  |  |
| End of treatment | 7.35 | 6.15 | 0.080 |
|  | (5.90, 9.98) | (4.09, 8.28) |  |

AES, the acupuncture expectancy scale; C-MASS, the Chinese version of the modified Massachusetts General Hospital Acupuncture Sensation Scale; SA, sham acupuncture; VA, verum acupuncture. *, Mann-Whitney U test was applied for the comparisons between VA and SA. A P value < 0.05 was considered statistically significant.
